# Breaking the age-related redox spiral for regeneration

**DOI:** 10.4103/NRR.NRR-D-25-01439

**Published:** 2026-02-05

**Authors:** Gregory J. Brewer, Bethany Cheung

**Affiliations:** Biomedical Engineering, University of California Irvine, Irvine, CA, USA; Institute for Memory Impairments and Neurological Disorders (MIND), Center for Neurobiology of Learning and Memory (CNLM), University of California Irvine, Irvine, CA, USA

Aging neurons do not fail randomly; rather, they enter a self-reinforcing redox spiral, rooted in metabolic responses to their environment, which diminishes their bioenergetic capacity. While pH measures proton donor potential, the redox state reflects electron donor potential, providing oxidative (e.g., NAD^+^) or reductive (NAD(P)H) power for numerous biochemical reactions. The decline in neuronal energy is orchestrated by mitochondrial checkpoints, transcriptional and post-transcriptional changes (Kumar et al., 2018), and proteostatic stress, all contributing to reduced synaptic resilience. Flux-control experiments highlight bottlenecks in the mitochondrial respiratory chain, particularly substrate-limited complex I and diminished capacity at complex IV (Jones and Brewer, 2010), resulting in increased electron leak, a more oxidized NAD^+^/nicotinamide adenine dinucleotide (NADH) and glutathione disulfide/glutathione (GSH) state, and a more oxidized quinone pool. Both young and old cortical neurons can increase respiration if provided with excess mitochondrial substrates, but old neurons are more sensitive to inhibition at complex IV. The decreased capacity for adaptive energy generation and electron transport through complex IV compared to young neuronal mitochondria likely results from regulatory nitrosylation by nitric oxide synthase (Torres et al., 1998). Importantly, aged neurons are especially dependent on endogenous substrate availability at complex I, suggesting that a shortage of NADH redox equivalents is a key constraint. This promotes glycolytic and epigenetic compensation (Walker et al., 2013), activating redox-sensitive programs that lock in an oxidative shift. Over time, the ability of the system to revert to a reduced, energy-efficient state narrows, making neurons vulnerable under metabolic stress and priming Alzheimer’s disease (AD)-related pathologies. This constraint is exacerbated in AD by decreased activity of dehydrogenases within the Krebs cycle that would lessen NADH production from NAD (Bubber et al., 2005).

According to the epigenetic oxidative redox shift theory (Brewer, 2010), the system locks into a destructive cycle when mitochondrial NADH oxidation fails and redox-sensitive transcription factors increase aerobic glycolysis to maintain adenosine triphosphate (ATP). Sustaining glycolytic ATP with impaired mitochondrial NADH-oxidoreductase requires auxiliary oxidoreductases to produce NAD(P)H (e.g., NADPH plasma membrane oxidoreductase), which efficiently generates damaging reactive oxygen species (ROS). Cells buffer damage by converting pyruvate to lactate, trading redox stress for lactic acidosis and further disconnecting glycolysis from oxidative phosphorylation. Over time, this shift is enforced by epigenetic regulation of genes coding for metabolic enzymes (Walker et al., 2013), and insulin resistance develops, reducing mitochondrial turnover (Gershon, 2001). The feedback loop escalates: oxidized redox state leads to more mitochondrial dysfunction, further oxidation, and decreasing energy capacity, increasing stress vulnerability. Control of this spiral centers on the NAD^+^/NADH system, which is upstream of GSH and the NADPH needed to regenerate GSH (Ghosh and Brewer, 2014b). Depleted NAD^+^ accelerates neurodegeneration in aging and AD model neurons (3×Tg-AD) more than GSH loss. The 3×Tg-AD model mouse has familial mutant APP genes that increase Aβ production and express a tau mutation seen in tauopathy. Decreased expression of key redox enzymes tracks with declining NADH, narrowing metabolic gates with age. As the redox state becomes more oxidized, the ability of cells to maintain a reduced cytosol, recycle antioxidants, and feed electrons to complex I diminishes, making downstream ROS markers a consequence, not the initial cause, of redox collapse.

This upstream collapse is visible in live neurons (Dong and Brewer, 2019b). Two-photon fluorescence lifetime imaging shows that mitochondrial-free NAD(P)H, which fuels complex I, decreases by about half in 3×Tg-AD model neurons compared to young controls, in contrast to unavailable NADH already bound to proteins. The deficit is not global; mitochondria remain more reduced than the cytosol, but their free NAD(P)H pool shrinks, likely due to NADH consumption as an antioxidant (Ghosh and Brewer, 2012), limiting input towards oxidative phosphorylation.

At the metabolomic level, mouse aging and the 3×Tg-AD-model Alzheimer’s genes lead to a coordinated remodeling of energy metabolism, especially at NAD^+^/NADH-dependent steps (Dong and Brewer, 2019a). Metabolites upstream of NAD^+^-dependent glycolytic and TCA cycle reactions decline, while downstream metabolites build up, indicating bottlenecks. Inflammatory lipids increase, neurotransmitter pools decline, and inputs to the electron transport chain from fatty acid oxidation and glutamine feed acetyl-CoA without stalling at redox gates. Since neurotransmitter pools and membrane lipids are downstream, restoring NAD redox balance could normalize synaptic transmitter synthesis and reduce inflammatory lipid production.

These constraints reshape our understanding of AD-related proteostatic pathology. In the 3×Tg-AD model, brain GSH levels decline before amyloid-beta (Aβ) accumulation and plaque deposition in the CA1 region (Pontrello et al., 2022). The pAkt/tAkt ratio, a marker of metabolic signaling, falls before plaques but after GSH, especially in the CA1. Importantly, providing a more reduced redox environment externally reduces intraneuronal Aβ in middle-aged neurons. Breaking the spiral requires interventions that restore redox control upstream: replenishing free NADH for complex I, increasing complex IV capacity, and resetting redox-sensitive transcriptional programs to reverse proteostatic failure.

**Reversing the aging energy crisis:** Since the redox spiral begins with NADH shortage and oxidative stress, the goal is not merely to raise ATP, but to normalize the demand-driven electron flow through complex I, reestablish a redox environment that reverses maladaptive glycolysis, reactivate mitonuclear communication, decrease ROS, and restore mitochondrial turnover. Augmenting NAD^+^ to raise mitochondrial free NADH is key: NAD(P)H is a stronger determinant of neuronal survival than GSH (Ghosh and Brewer, 2014b). Thus, replenishing NAD^+^ precursors via the nicotinamide salvage pathway and transhydrogenase fuels complex I and regenerates GSH while supporting reductive biosynthesis using NADPH. Elevating free mitochondrial NADH increases electron flux, and a balanced ubiquinone pool reduces pressure at complex III, lowering ROS. However, increased electron transport and Krebs cycle activity generate ROS as a byproduct, which can cause damage.

Therefore, combining an NAD^+^ precursor (nicotinamide) with an Nrf2 inducer (many phytochemicals such as epigallocatechin gallate from green tea) induces a suite of redox enzymes (NQO1, GST, TrxR, malic enzyme, HO1, GCLC, and catalase) to lower ROS. Subcellular measurements show that manipulating the extracellular cysteine/cystine redox couple with excess reductive cysteine restores mitochondrial free NAD(P)H in old neurons to youthful levels (Dong et al., 2019b), indicating cysteine as a limiting factor for GSH synthesis in aged and 3×Tg-AD-model neurons. This also suggests neurons communicate across the plasma membrane, linking extracellular thiol-disulfide status to intracellular NAD(P)H pools and mitochondrial function. Resetting to a more reduced extracellular potential relieves intracellular redox stress, lowers ROS, and increases survival signaling, suggesting the redox environment itself determines energy allocation.

Direct experimental evidence for this upstream redox framework comes from combined NAD+ replenishment and Nrf2 activation that yields greater neuroprotection than either alone (Ghosh and Brewer, 2014a). For example, pairing nicotinamide with 18α-glycyrrhetinic acid, a natural Nrf2 activator from licorice, synergistically boosts glutathione levels and neuronal survival in aging and 3×Tg-AD-model neurons. This dual strategy directly replenishes NADH for complex I and NADPH for GSH regeneration, while Nrf2 activation upregulates glutathione synthesis and the rate-limiting enzyme γ-glutamylcysteine synthetase.

Restoring a reduced redox state also promotes proteostasis. Autophagy and endolysosomal trafficking rely on local guanosine triphosphate (GTP) pools and the function of Rab and Arl family GTPases, which manage vesicle formation, transport, and degradation. Aging neurons have lower free GTP, especially in mitochondria, impairing vesicular energetics and stalling autophagy needed to clear damaged proteins and lipids, including aggregated peptide Aβ (Santana et al., 2025). Supplementing aged neurons with an NAD precursor and a redox modulator restored GTP levels, reactivated vesicular GTPases, accelerated autophagic clearance to lower intracellular Aβ aggregates, and improved neuronal viability. Thus, boosting NAD redox capacity and activating a more balanced redox state together rebuilds vesicular energy budgets, allowing the autophagy machinery to relieve proteostatic stress and break the redox spiral.

**Figure 1:**
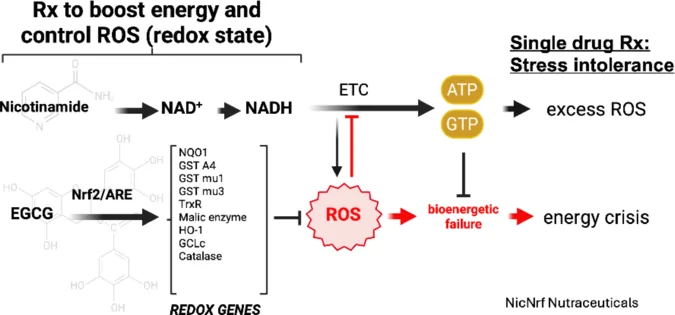
Combination of nicotinamide and EGCG boosts NAD^+^ and Nrf2 for better energetic and redox control. Greater efficacy of the combination of an NAD^+^ redox energy precursor, nicotinamide, with green tea catechin EGCG to induce transcription factor Nrf2 for redox state (ROS) control than either alone. Created with BioRender.com. ARE: “anti-oxidant” redox response element; EGCG: epigallocatechin-3-gallate; ETC: mitochondrial electron transport chain; NAD^+^: oxidized nicotinamide adenine dinucleotide; NADH: reduced nicotinamide adenine dinucleotide; Nrf2: nuclear factor erythroid 2-related factor 2; ROS: reactive oxygen species; Rx: drug treatment.

Three principles guide reversal without causing reductive stress: (1) Raising NAD^+^ must be paired with adequate complex IV capacity and oxygen handling to prevent NADH buildup and unwanted superoxide generation or excess lactate (**Figure 1**). In aging, complex IV reserve is lowered; interventions that enhance cytochrome c oxidase (such as reduced sugar intake, exercise, and cognitive stimulation) can expand capacity and lower redox pressure. (2) Because extracellular Cys/CySS dictates intracellular redox tone, modest external reduction can reset intracellular NAD(P)H and GSH. Increasing NADH and activating Nrf2 promote full nucleotide pools and re-energize GTPases for autophagy and endolysosomal throughput, enabling the removal of damaged mitochondria and protein aggregates. (3) Overall, reversal involves restoring cellular homeostasis with balanced redox, healthy mitochondrial NAD(P)H, sufficient complex IV capacity, and functional GTP-dependent trafficking. This enables recoupling of glycolysis and oxidative phosphorylation, restoring transmitter synthesis and adaptive plasticity.

**Benefit from combination of redox modulator and energy precursor:** Combination therapy is mechanistically logical, not just additive: an NAD^+^ precursor fills the energetic “fuel line,” while a redox modulator resets control logic and detoxification, restoring not just ATP but the redox conditions for sustainable electron flow and proteostatic health (**Figure 1**). Pairing nicotinamide with an Nrf2 activator such as epigallocatechin gallate yields additive neuroprotection in aging and in the 3×Tg-AD-model neurons by improving survival under Aβ stress (Ghosh and Brewer, 2014a), elevating GSH, normalizing a broad array of redox enzymes. These studies were recently extended to the Denali APP knock-in mouse model, APP^SAA^ (Santana and Brewer, unpublished). This synergy extends to vesicular energetics and clearance. The combination restores GTP, reactivates vesicular GTPases, resumes autophagy, lowers Aβ aggregates, and reduces oxidized biomolecules. Improved NAD(P)H and GSH reduce oxidative pressure, stabilizing mitochondria to maintain GTP synthesis, which further restores autophagic clearance and lowers redox stress. Viewed through EORS (Epigenetic Oxidative Redox Shift theory of aging) (Brewer, 2010), this combination breaks a vicious cycle to restore youthful metabolism.

However, translating *in vitro* combination therapy to clinical use requires attention to pharmacokinetics and tissue targeting. High-dose oral nicotinamide in the human NEAT trial massively raised plasma levels but also results in even higher levels of inactive metabolites such as methyl-nicotinamide and limited brain availability (Ketron et al., 2025). Less than a third of patients showed central nervous system delivery, but they experienced 34% declines in pathological markers such as pTau231 over 48 weeks. This indicates that the efficacy of nicotinamide is limited by delivery and inactivation, but combination strategies may mitigate this. Adding a redox modulator may enhance neuronal redox poise and detoxification, lowering the required NAD^+^ precursor dose and reducing metabolic burden. Adjusting the extracellular redox environment (e.g., shifting the Cys/CySS couple) further boosts intracellular NAD(P)H and GSH without high drug levels.

The value of combination therapy lies in strategically coupling interventions to restore coordinated physiological states. This flexibility lets neurons meet synaptic demands without reverting to excessive glycolysis or oxidant production. For translation, the field should align on quantitative benchmarks such as plasma or red cell mitochondrial NAD(P)H, NADH/NAD^+^ ratios, GSH/glutathione disulfide, plasma Aβ_42_/tau217 ratios, complex IV reserve metrics, and brain Aβ burden by PET imaging. Early trials can reduce false negatives by stratifying patients based on levels of nicotinamide inactivation following test dosing. Alternative routes of delivery, such as intranasal or intravenous injection, should also be considered due to extensive methylation and restricted CNS penetration following oral administration (Ketron et al., 2025). Combined metabolic redox activators also looked promising for cognitive improvement in a double-blinded, placebo-controlled phase-II trial in AD subjects (Yulug et al., 2023).

In summary, the energy crisis of aging brain is addressable when viewed as a redox control problem with interconnected energy systems. By restoring upstream electron supply, redox balance, and by re-energizing vesicular clearance, a combination of energy precursors and redox modulators can not only halt but reverse neurodegeneration, reopening the window for synaptic plasticity and cognitive resilience.

*This work was supported by the UC Irvine Foundation to GJB.*
